# Comparative Analysis of pH and Target-Induced Conformational Changes of an Oxytetracycline Aptamer in Solution Phase and Surface-Immobilized Form

**DOI:** 10.3390/biom13091363

**Published:** 2023-09-07

**Authors:** Kristóf Jakab, Nikitas Melios, George Tsekenis, Abdul Shaban, Viola Horváth, Zsófia Keresztes

**Affiliations:** 1 Functional Interfaces Research Group, Institute of Materials and Environmental Chemistry, Research Centre for Natural Sciences, Magyar tudósok krt. 2, 1117 Budapest, Hungary; shaban.abdul@ttk.hu; 2 Department of Inorganic and Analytical Chemistry, Faculty of Chemical Technology and Biotechnology, Budapest University of Technology and Economics, Műegyetem rkp. 3, 1111 Budapest, Hungary; horvath.viola@vbk.bme.hu; 3 Biomedical Research Foundation, Academy of Athens, 4 Soranou Ephessiou Street, 115 27 Athens, Greece; nmelios@bioacademy.gr (N.M.); gtsekenis@bioacademy.gr (G.T.); 4 ELKH-BME Computation Driven Chemistry Research Group, Műegyetem rkp. 3, 1111 Budapest, Hungary

**Keywords:** aptamer, single-stranded DNA, G-quadruplex, oxytetracycline, circular dichroism, quartz crystal microbalance

## Abstract

To date, numerous aptamer-based biosensing platforms have been developed for sensitive and selective monitoring of target analytes, relying on analyte-induced conformational changes in the aptamer for the quantification of the analyte and the conversion of the binding event into a measurable signal. Despite the impact of these conformational rearrangements on sensor performance, the influence of the environment on the structural conformations of aptamers has rarely been investigated, so the link between parameters directly influencing aptamer folding and the ability of the aptamer to bind to the target analyte remains elusive. Herein, the effect a number of variables have on an aptamer’s 3D structure was examined, including the pH of the buffering medium, as well as the anchoring of the aptamer on a solid support, with the use of two label-free techniques. Circular dichroism spectroscopy was utilized to study the conformation of an aptamer in solution along with any changes induced to it by the environment (analyte binding, pH, composition and ionic strength of the buffer solution), while quartz crystal microbalance with dissipation monitoring was employed to investigate the surface-bound aptamer’s behavior and performance. Analysis was performed on an aptamer against oxytetracycline, serving as a model system, representative of aptamers selected against small molecule analytes. The obtained results highlight the influence of the environment on the folding and thus analyte-binding capacity of an aptamer and emphasize the need to deploy appropriate surface functionalization protocols in sensor development as a means to minimize the steric obstructions and undesirable interactions of an aptamer with a surface onto which it is tethered.

## 1. Introduction

Aptamers are relatively short (~15–75 bases), single-stranded oligonucleotides (DNA or RNA) that bind selectively and with high affinity to their respective targets, against which they are artificially selected through the process of Systematic Evolution of Ligands by Exponential Enrichment (SELEX) [1,2]. During this procedure, a random oligonucleotide library is incubated with the target. The unbound sequences are washed away, while the bound oligomers are eluted and amplified via polymerase chain reaction (PCR). These steps are usually repeated 10 to 20 times with increasingly stringent conditions employed in each round, resulting in the selection of aptamer sequences with enhanced affinity to a target analyte. [3]. Counter selection is also often employed, during which sequences interacting with undesired targets (similar molecules, matrix elements, etc.) can be excluded from the pool of the random oligonucleotides. The resulting aptamer sequences hold great promise in substituting antibodies as biorecognition elements due to their improved stability, simpler and more inexpensive production and the ease with which site-specific chemical modification can be introduced. For these reasons, they have been commonly employed in biosensors coupled with a diverse range of signal transduction principles, such as colorimetric [4], fluorimetric [5,6], electrochemical [7,8] and acoustic [9,10].

Although significant progress has been made in the development of aptamer-based sensors and their deployment in a diverse set of applications, little is known about the ways with which they interact and bond with their target analytes, and their conformation prior to and upon binding to a target analyte is still the subject of intense debate. Despite the key roles that the knowledge of an aptamer’s 3D structure and any analyte-induced changes to its conformation play to the rational design of aptamer-based assays, there are little data on aptamer structural motifs, while there are even fewer resolved and well-characterized aptamer-target analyte complexes. The scarcity of relevant information hinders the development of robust aptamer-based assays, as well as the integration of assays into new sensing platforms that could potentially enhance measurement sensitivity, both of which ultimately prevent aptamers from realizing their full potential [11,12]. Furthermore, the effect that the aptamer immobilization onto a solid support, such as a sensor surface, has on its folding has not been adequately examined, despite the fact that the retainment of an aptamer’s native conformation is crucial to its ability to recognize and interact with its target analyte. The focus of most of the published work has been the enhancement of the performance of electrochemical aptamer-based sensors, providing little insight into how the aptamer itself is affected upon tethering to a surface [13]. Moreover, most of the work has been undertaken with the use of the thrombin-binding aptamers [14,15,16], with hardly any data being available on aptamers against small-molecule (<1 kDa) analytes [17]. Therefore, gaining insight into the impact of immobilization, or generally speaking, the environment of an aptamer (temperature, pH) on its conformation directly, without the need for tagging, is of paramount importance and especially so for aptamers whose targets are of low molecular weight. Nevertheless, only a limited number of studies present data on the structure of an aptamer in solution, as well as following its immobilization onto a solid support. To this avail, circular dichroism spectroscopy (CD) complemented by quartz crystal microbalance (QCM) measurements are ideally-suited for this purpose.

Circular dichroism spectroscopy is an analytical method used to examine optically active chiral molecules by measuring the differential absorption of left- and right-handed polarized light. Individual nucleotides are not chiral themselves, but oligonucleotides do present a CD spectrum as an outcome of base stacking and the secondary and tertiary structures that nucleotide sequences conform to. CD spectra of DNA have been empirically assigned to the A-, B- and Z-form of the molecule, as well as some well-defined tertiary structures such as the G-quadruplex, the cytosine-rich *i*-motif and also to stem–loop motifs [18,19,20,21]. Nevertheless, CD spectroscopy has been rarely employed to follow the conformational transitions of an aptamer upon exposure to different buffer solutions or buffers with varying ionic compositions and pH values.

Quartz crystal microbalance is an acoustic technique that measures changes to the resonance frequency of a piezoelectric quartz crystal resonator (QCR) due to the addition or removal of mass on the surface. Dissipation (D) monitoring is equally important, as it provides information on the viscoelastic properties of the adlayer, as well as any alteration induced by the level of hydration or structural rearrangements of the surface-tethered biomolecules themselves [22]. Numerous studies have been published that include the interrogation of aptamer-modified surfaces via QCM with a dissipation mode. Nevertheless, most of the investigations have focused on the use of aptamers for the detection of proteins and/or pathogens, whose comparatively large mass facilitates analysis and allows large signal responses to be obtained [23]. By contrast, studies on aptamer-based monitoring of small molecule analytes with QCM are very limited, since the binding of the latter results in frequency changes of only few Hz, leading to low signal-to-noise ratios [24,25,26].

Herein, in an attempt to gain a better understanding on the effect of the solution environment as well as the surface immobilization on the ability of an aptamer to fold and recognize its target analyte, an aptamer against oxytetracycline (OTC) was employed as a model system. OTC, a member of the tetracycline family of antibiotics, was chosen due to its widespread and excessive use in livestock, which has been linked to the increasing emergence of antibiotic-resistant bacteria [27]. Developing reliable analytical sensor systems for OTC detection in various environmental and food matrices is therefore of paramount importance, and this is why a large number of biosensors for OTC detection have already been developed, including numerous aptasensors [28,29,30]. The performance of such OTC sensors and by inference the general performance of aptasensors against other small-molecule analytes can be improved by investigating how an OTC aptamer folds in response to OTC, as well as a number of other factors. The aptamer sequence utilized in this work was selected by Niazi and coworkers [31], and although it has already been extensively used in the construction of sensors [6,7,8,9,32,33,34], its conformation as well as the effect of buffer pH, target binding and surface immobilization have never been studied before. Towards this goal, CD spectroscopy and QCM with dissipation measurements were employed, which are complementary techniques suitable for the interrogation of aptamers in solution and surface-bound form.

## 2. Materials and Methods

### 2.1. Chemicals

Sodium chloride, potassium chloride, magnesium chloride hexahydrate, calcium chloride dihydrate, disodium hydrogen phosphate, dipotassium hydrogen phosphate, tris(hydroxymethyl)aminomethane (Tris) base, trisodium citrate, Tween 20, oxytetracycline hydrochloride, oxytetracycline dihydrate and tris(2-carboxyethyl)phosphine (TCEP) were purchased from Sigma-Aldrich, Budapest, Hungary. Paraffin oil was obtained from Metallex Group, Athens, Greece, while potassium hydroxide and l-cysteine were purchased from Reanal, Budapest, Hungary. Moreover, 30% hydrogen peroxide was purchased from Lach-Ner, Neratovice, Czech Republic. LGC Biosearch Technologies, Lystrup, Denmark, and Integrated DNA Technologies, Leuven, Belgium supplied the DNA aptamers for the CD measurements. The sequences were as follows: 5′-GGA ATT CGC TAG CAC GTT GAC GCT GGT GCC CGG TTG TGG TGC GAG TGT TGT GTG GAT CCG AGC TCC ACG TG-3′ (71-mer) and 5′-ACG TTG ACG CTG GTG CCC GGT TGT GGT GCG AGT GTT GTG T-3′ (40-mer). The thiol-modified 40-mer aptamer for the QCM measurements was supplied by LGC Biosearch Technologies, Lystrup, Denmark with the following sequence: 5′-ACG TTG ACG CTG GTG CCC GGT TGT GGT GCG AGT GTT GTG T-(CH_2_)_6_-S-S-(CH_2_)_6_-OH-3′.

### 2.2. Buffer Preparation

The Tris binding buffer contained 20 mM Tris, 100 mM NaCl, 5 mM KCl, 2 mM MgCl_2_ and 1 mM CaCl_2_. A similar buffer was used in the binding process of the SELEX procedure of the aptamer [31]. For the experiments, phosphate-buffered saline (PBS) (10 mM Na_2_HPO_4_, 1.8 mM K_2_HPO_4_, 150 mM NaCl, 2.6 mM KCl) and saline-sodium citrate (SSC) (150 mM NaCl, 1.5 mM trisodium citrate) buffers were also made. The K^+^, Mg^2+^ and Ca^2+^ ion contents and the pH of the buffers were adjusted prior to the respective experiments. PBS buffer pH was adjusted in the range of 7.1–9.1 and the SSC buffer in the pH range of 3.1–6.6, with 0.5 pH increments in both cases. In the SSC buffer, the K^+^, Mg^2+^ and Ca^2+^ ions were used in the same concentrations as in the Tris binding buffer. For the investigation of the effect of ionic content, the following media were used: ultrapure water, PBS, Tris binding buffer and Tris buffer containing K^+^, Mg^2+^ and Ca^2+^, either at the concentration of the binding buffer or none, in every possible variation. Moreover, 100 mM sodium was present in all Tris buffer solutions. The buffer solutions were filtered using 0.20 µm or 0.22 µm syringe filters and sterilized if their storage time exceeded 5 days.

### 2.3. Circular Dichroism Spectroscopy Measurement

CD measurements were performed on a Jasco J-1500 CD Spectrometer in photometric mode. The measurement range was 200–320 nm, with 2 nm bandwidth and spectra taken at 50 nm/min scanning speed during the OTC titration experiments. The sample holder was thermostated at 25 °C. Buffer solutions without any aptamer or OTC were used for baseline correction. The denaturation measurements were performed without baseline correction. The scanning speed was set at 100 nm/min, the temperature was increased at a rate of 2 °C/min between 4 and 100 °C and spectra were taken at every 2 °C.

For titration, 500 µL of 5 µM aptamer solution was pipetted into a 5 mm quartz cuvette. An aqueous solution of OTC was pipetted into the aptamer solution to obtain concentrations of 2.5, 5, 10, 20 and 40 µM. Before the CD measurement, the solutions were gently shaken and incubated at room temperature for 5 min.

Denaturation experiments were carried out using 1000 µL of 5 µM aptamer solution alone and with 40 µM OTC in a 5 mm quartz cuvette with a PTFE lid. In addition, 100 µL paraffin oil was layered on top of the solution to minimize evaporation.

### 2.4. Quartz Crystal Microbalance Measurement

The QCM with an impedance analysis (QCM-I) unit (MicroVacuum Ltd., Budapest, Hungary) was controlled via BioSense 3 software (MicroVacuum Ltd., Budapest, Hungary. The QCM-I device’s resonance and dissipation sensitivity in liquid are 0.2 Hz and 1 × 10^−7^, respectively. Solutions were drawn through the QCM fluidic cell using an Ismatec peristaltic pump at 60 µL/min. To change solutions, the pump was stopped for a moment, the tubing was immersed into the next solution and the pump was started again. The cell temperature was thermostated to 25 °C. Besides the fundamental resonance frequency, dissipation was also registered at odd-numbered overtones up to the 13th.

The dithiol-modified aptamer solution was incubated with TCEP at room temperature for 1 h to reduce the dithiol to the more reactive thiol. It was then diluted to 1 µM, incubated in a Grant bio PCH-1 Dry-block at 95 °C for 5 min and allowed to cool to room temperature so that the aptamer assumes its native conformation after being stored frozen.

QCRs, 14 mm, 5 MHz Ti/Au (MicroVacuum Ltd., Budapest, Hungary), were first cleaned with a 50 mM KOH-25% H_2_O_2_ solution [35], then rinsed with ultrapure water and dried using compressed air flow. The gold surface of the QCRs was modified by dropping 150 µL of the following solutions: (I) 1 µM aptamer solution; (II) 0.1 µM l-cysteine solution; (III) 1 µM aptamer solution followed by 0.1 µM l-cysteine solution (low-density coverage); and (IV) 10 µM aptamer solution followed by 0.1 µM l-cysteine solution (high-density coverage). The solutions were left on the crystal for 1 h, rinsed with ultrapure water and then immersed into 7.1 pH Tris binding buffer until measurement.

## 3. Results and Discussion

### 3.1. Induced Conformational Changes in Solution Phase

**Figure 1:**
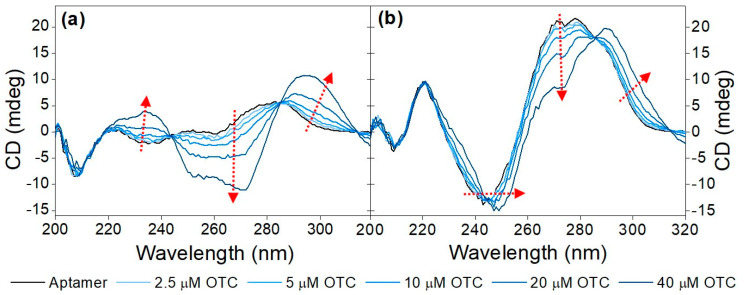
Target-induced conformational change of (**a**) 40-mer aptamer (5 µM) and the (**b**) 71-mer aptamer (5 µM) in Tris binding buffer (pH = 7.6) with increasing concentrations of OTC (2.5 μM, 5 μM, 10 μM, 20 μM and 40 μM). The red arrows point in the direction of increasing concentration.

Initially, the CD spectra of the variable region of the aptamer including the primers flanking it (71-base-long) and without them (40-base-long) were recorded in the absence as well as in the presence of increasing OTC concentrations (Figure 1). As both sequences contain a high number of guanine bases, it was anticipated that they assume a guanine quadruplex (G-quadruplex) structure. G-quadruplexes are made up of multiple G-quartets, planar square motifs formed by four guanine bases. G-quadruplexes can be categorized into three main subtypes: parallel, antiparallel and hybrid [20,21,36,37]. Parallel G-quadruplexes can be identified by their large positive peak at 260 nm, and a shallow negative one at 240 nm. Antiparallel G-quadruplexes, on the other hand, display a negative peak at 260 nm and a positive peak at around 295 nm, while the peaks of the hybrid structures fall between the peaks of the previous two, showing positive peaks at 295 nm and 260 nm, as well as a negative one at 240 nm. These wavelength values are averages of the values reported in the literature; the exact values where peaks are formed are influenced by differences in G-quartet stacking, strand segment orientation and loop arrangements [38]. This shows the ambiguity when interpreting CD spectra of G-quadruplex structures.

Analysis of the CD spectra obtained for the two sequences shows some common structural features, such as the negative peak at ca. 205 nm, which is characteristic of GC-rich sequences adopting an A form even in aqueous solutions [18]. The rest of the structural elements that can be deduced from the recorded spectra differ significantly between the two sequences. For the 40-mer, no distinctive features can be deduced in the absence of the analyte, whereas the CD spectrum of the 71-mer indicates that it adopts a parallel G-quadruplex. Incubation with OTC results in the adoption of the most thermodynamically favorable structure, which is different for the two sequences under examination. As far as the 40-mer is concerned, the increase at ca. 230–240 nm, the decrease at ca. 250 nm–270 nm and the increase at ca. 290 nm (as indicated by the red arrows) observed in the presence of increasing concentrations of OTC is indicative of the analyte-induced formation of an antiparallel G-quadruplex. In the case of the 71-mer, increasing OTC concentrations result in a shift in the minimum peak from ca. 240 nm to ca. 250 nm, coupled with a decrease in the maxima at ca. 270 nm and the appearance of a peak at ca. 290 nm. Once again, and as was the case for the 40-mer, the shifts in the spectra observed for the 71-mer are analyte-induced and most probably testify to the folding of the aptamer into a hybrid G quadruplex or a mixture of hybrid and antiparallel quadruplexes. For practical purposes, and since both sequences show binding to the analyte, the shorter 40-mer sequence was selected in all subsequent experiments.

**Figure 2:**
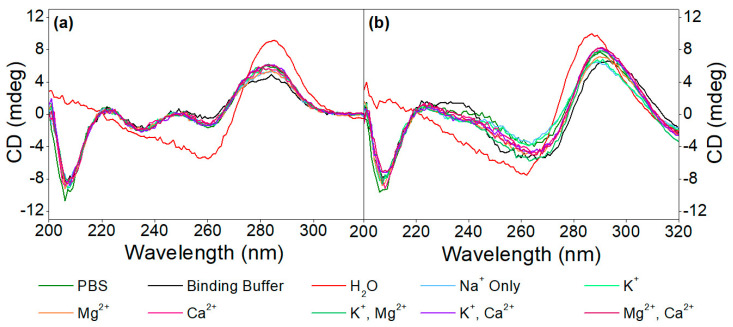
CD spectra of (**a**) 5 µM aptamer and (**b**) 5 µM aptamer in the presence of 20 µM OTC in different media, as well as in Tris buffer, in the presence of various monovalent and divalent cations.

Subsequently, the effect of different cations (K^+^, Mg^2+^, Ca^2+^ or combinations thereof) on the aptamer structures, as well as on the target-induced conformational changes, was examined in different media, as seen in Figure 2. The CD spectra of the 40-mer aptamer recorded in the absence of the analyte revealed that they conform to the same structure, irrespective of the ionic composition or strength of the buffer, which also holds true for the buffering media employed, while in the complete absence of ions (i.e., in pure H_2_O), different conformation was observed. In other words, the aptamer folds into its respective structure simply in the presence of monovalent cations, while divalent cations do not significantly influence this structure, which is not the case with other G-quadruplex conforming aptamers, whereby the presence of divalent cations is of paramount importance for their folding [21,39]. Nonetheless, when the buffer used during the SELEX process is employed, and therefore the aptamer is in the presence of both monovalent (Na^+^ and K^+^) as well as divalent cations (Mg^2+^ and Ca^2+^), the CD spectra recorded show similar shifts to those induced by the analyte itself, unlike in pure H_2_O. This is why the binding buffer was employed in all subsequent experiments.

#### 3.1.1. Thermal Denaturation Study

Thermal denaturation or DNA melting of the 40-mer on its own, as well as in complex with OTC, was performed to establish whether the analyte has a stabilizing effect on the aptamer. Raising the temperature by 2 °C increments results in gradual breakage of the hydrogen bonds and the hydrophobic stacking between the bases; both are responsible for maintaining the 3D conformation of the aptamer. Above a certain temperature, the native conformation of the aptamer is lost completely, and the oligonucleotide exists as a random coil. This point will depend on the stability of the oligonucleotide sequence based on the number and strength of interactions between its bases and will be affected by the new bonds that form between the aptamer and its analyte [40,41].

**Figure 3:**
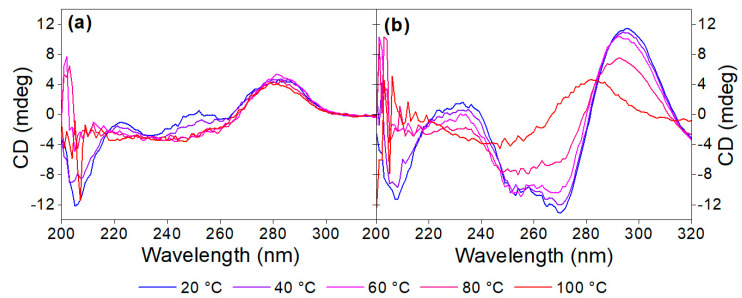
Denaturation of (**a**) 5 µM aptamer and (**b**) 5 µM aptamer in the presence of 40 µM OTC in Tris binding buffer pH = 7.1.

**Figure 4:**
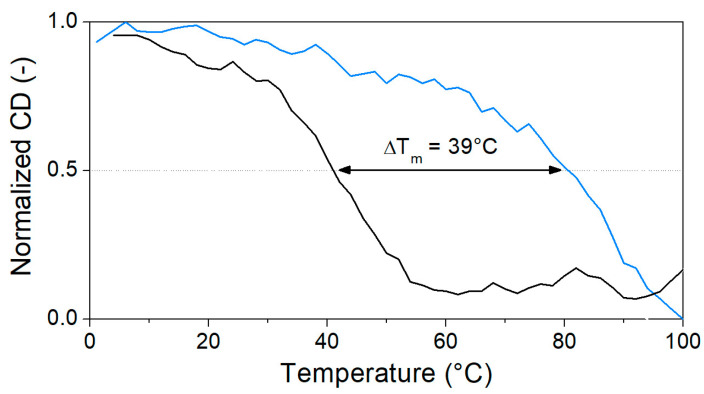
Normalized CD change in characteristic peaks of unbound (black, 250 nm) and OTC-bound (blue, 270 nm) aptamers through the denaturation process (5 µM aptamer and 40 µM OTC in Tris binding buffer pH = 7.1).

Heat denaturation of the aptamer resulted in small changes in the recorded CD spectrum, reaffirming the lack of prominent structural motifs in the 40-mer in the absence of its analyte. The decrease in the peaks at ca. 220 and 250 nm, as well as the disappearance of minima at ca. 205 nm, are indicative of the stacking between the bases and the accompanying increased helicity (Figure 3a). As 40 µM OTC is introduced (Figure 3b) at low temperature, the positive peak at 280 nm shifts to 295 nm, and a negative peak appears around 260 nm, indicating binding of the aptamer to its target. However, as the temperature increases, the two peaks are diminishing in intensity, indicating decreased binding. For the determination of T_m_, we identified two wavelengths, where the CD absorbance changes the most during denaturation, 250 nm for the native aptamer and 270 nm for the aptamer–target complex. The CD changes were then normalized and plotted together as a function of the temperature (Figure 4). From the curves, the melting temperatures could be obtained. The T_m_ of 41 °C for the native aptamer increased to 80 °C upon binding with OTC. This convincingly shows the structure-stabilizing effect of target binding.

#### 3.1.2. Effect of pH

The effect of pH on DNA conformation has been primarily investigated for nanoswitches, as well as in drug-delivery and pH sensor applications, focusing on its effect on distinctive structures such as the cytosine-rich *i*-motif and G-quadruplexes [42,43,44,45,46]. However, it has rarely been investigated with regards to surface-confined aptamers and aptasensors, even though the pH can greatly affect the DNA conformation through the charge incurred on DNA bases and on the target itself [47,48,49].

To investigate the effect of pH on the OTC aptamer, CD spectra were acquired in the pH range of 3.1–9.1, using Tris buffer for the alkaline pH range (7.1–9.1) and SSC buffer for the acidic range (3.1–6.6). K^+^, Mg^2+^ and Ca^2+^ ions were used in the same concentrations as in the Tris binding buffer.

**Figure 5:**
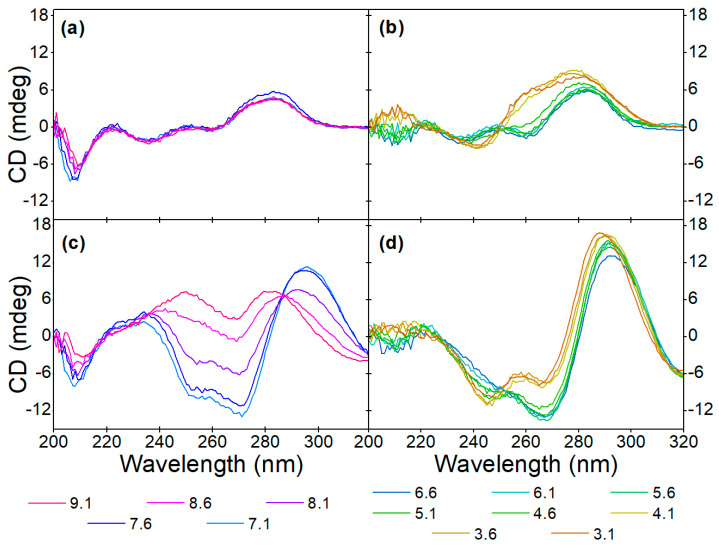
Effect of pH on the aptamer conformation at (**a**,**b**) 0 µM and (**c**,**d**) 40 µM OTC concentrations in (**a**,**c**) alkaline and (**b**,**d**) acidic solutions.

The pH-dependent target-induced changes in the alkaline pH range can be evaluated from the CD spectra shown in Figure 5a,c. While the conformation of the native aptamer is the same at all five adjusted pH values, the binding of OTC facilitates different structural changes. Up to 8.1 pH, the characteristic peaks of the antiparallel G-quadruplex are discernible, but in the more alkaline solutions, positive peaks around 250 nm and 280 nm appear. We hypothesize that the deprotonation of the guanine bases’ N1 nitrogen prevents quadruplex formation, considering that this hydrogen is involved in half of the hydrogen bonds in the G-quadruplex structure [50]. However, since no previous work has been conducted regarding this, further investigations are needed to confirm our theory.

In the acidic pH range (Figure 5b,d), however, the unbound aptamer shows different behavior. When the pH is below 4.6, a negative peak at 240 nm and a positive peak at 260 nm appears, and the positive peak at 290 nm increases in intensity. According to the literature, this is due to the protonation of cytosine bases [51]. This structure, contrary to observations in alkaline conditions, seems to be undisturbed by the OTC interaction, i.e., the deviations from the CD spectra of the OTC-bound aptamer at pHs higher than 4.1 at the wavelengths of 240 nm, 260 nm and 290 nm remain the same. While the CD spectra of the native aptamer in the acidic pH resemble hybrid G-quadruplex conformation, the fact that the pH-induced structure is unaffected by the OTC addition suggests that this structure is most likely in a different part of the DNA strand than the sequence responsible for OTC binding.

**Figure 6:**
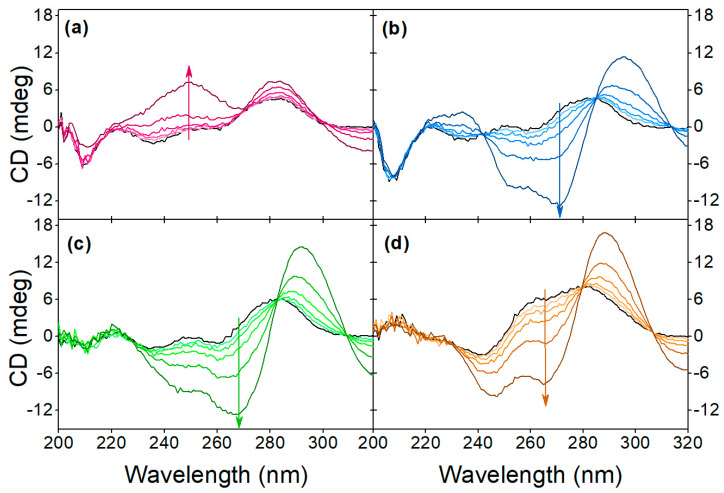
Effect of pH on the target-induced conformational change at (**a**) pH 9.1, (**b**) 7.1, (**c**) 5.1, (**d**) 3.1. The arrows indicate the increase in OTC concentration, the black line indicates the native aptamer and the OTC concentration values are 2.5 μM, 5 μM, 10 μM, 20 μM and 40 μM.

In Figure 6, the characteristic concentration-dependent target-induced conformational changes can be seen throughout the investigated pH range. The pH of the binding buffer affects the native conformation of the aptamer, as well as the aptamer-target interaction. The buffer type also seems to affect the CD spectra, most noticeably the intensity of the negative peak at 205 nm is greater in the case of Tris buffer compared to the SSC buffer. Since at neutral and acidic pH the sensitivity of the aptamer and the nature of the binding are unchanged, the charge of OTC can only have a relevant effect above pH 8.6.

### 3.2. Induced Structural Changes in Surface-Immobilized Aptamer

The behavior of the surface-bound aptamer in response to different pH and OTC concentrations was investigated using QCM with dissipation monitoring. We followed the frequency and dissipation change in the QCR on the third overtone, considering that the planar crystal sometimes behaves irregularly in the fundamental mode [22]. We assign the initial frequency shift to the hydration of modified QCR surface.

#### 3.2.1. pH-Induced Responses of the Aptamer

**Figure 7:**
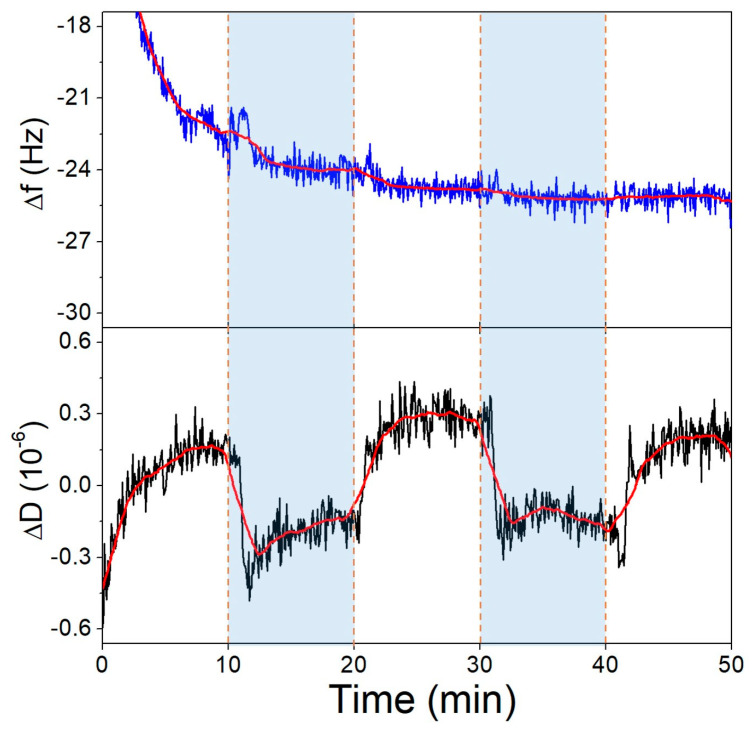
The third overtone frequency (**top**) and dissipation (**bottom**) variation values of the aptamer-modified gold surface in response to pH change. Dashed lines indicate solution changes, with white background corresponding to pH 6.6 and blue to pH 3.6.

First, we investigated the pH-induced conformational change of the surface-bound aptamer, where the thiolated 40-mer aptamer was deposited on the gold surface of the QCR and SSC buffer of pH 6.6 and pH 3.6 were exchanged over it, repeatedly. In Figure 7, it is shown that the dissipation decreases as the pH is lowered, which corresponds to the aptamer layer becoming more compact. Cytosine-rich sequences form a dense quadruplex structure called an *i*-motif in acidic pH due to the deprotonation of the bases. The formation of such a conformation explains both the CD (Figure 5b) and the QCM results. Supporting QCM results are already published regarding *i*-motif formation [52]. The frequency of the crystal during this measurement did not change due to differences in pH.

#### 3.2.2. Target-Induced Responses of the Aptamer

##### Non-Specific OTC Adsorption on Gold and l-Cysteine-Based Antifouling Layer

**Figure 8:**
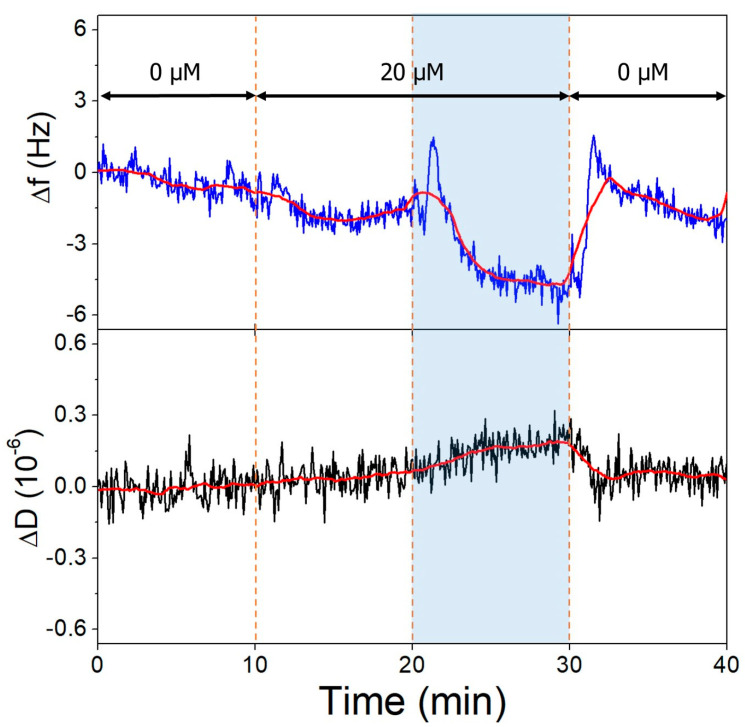
The third overtone frequency (**top**) and dissipation (**bottom**) variation values of clean gold surface in response to OTC adsorption. Dashed lines indicate solution changes, with white background corresponding to pH 7.1 and blue to pH 9.1. Different OTC concentrations are indicated in the figure.

Non-specific binding of OTC to clean gold QCR surfaces was observed upon switching from buffers to different pH OTC solutions (Figure 8). This interaction is pH-dependent, since at pH 9.1, a greater amount of OTC adsorbs onto the surface, than at pH 7.1. This is probably due to the charge state of the OTC molecule, being mostly neutral in a zwitterionic state at 7.1 and negatively charged at 9.1.

**Figure 9:**
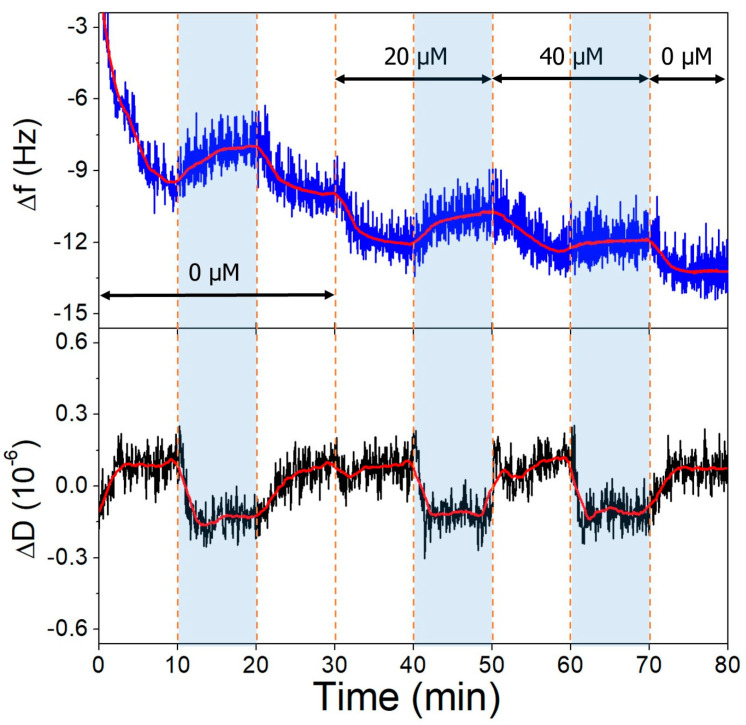
The third overtone frequency (**top**) and dissipation (**bottom**) variation values of l-cysteine-modified gold surface in response to OTC. Dashed lines indicate solution changes, with white background corresponding to pH 7.1 and blue to pH 9.1. Different OTC concentrations are indicated in the figure.

This target adsorption on gold is undesired when investigating aptamer-OTC binding; therefore, we deposited an l-cysteine antifouling layer on the gold after aptamer immobilization to prevent it [53,54]. This amino acid is zwitterionic in neutral pH, while at pH 9.1, the amine group becomes deprotonated (pK ≈ 8.2), resulting in a negatively charged surface layer, which repels the similarly negative OTC molecules. The antifouling layer succeeded in hindering the OTC adsorption at both investigated pH values (Figure 9); here, a slight baseline shift was observed. On the other hand, the increase in pH affected the l-cysteine layer and resulted in a dissipation decrease and a frequency increase on the QCR, which is due to the different hydration levels of the deposited layer, facilitated by the deprotonation of the amine group.

##### OTC-Aptamer Binding, the Effect of Surface Density and pH

The CD spectra showed that the conformational change induced by OTC titration was not affected by the pH in the acidic pH range, unlike in the alkaline range (Figure 4). Due to this, the effect of target binding on the surface-bound aptamers was only investigated in the pH range of 7.1–9.1.

**Figure 10:**
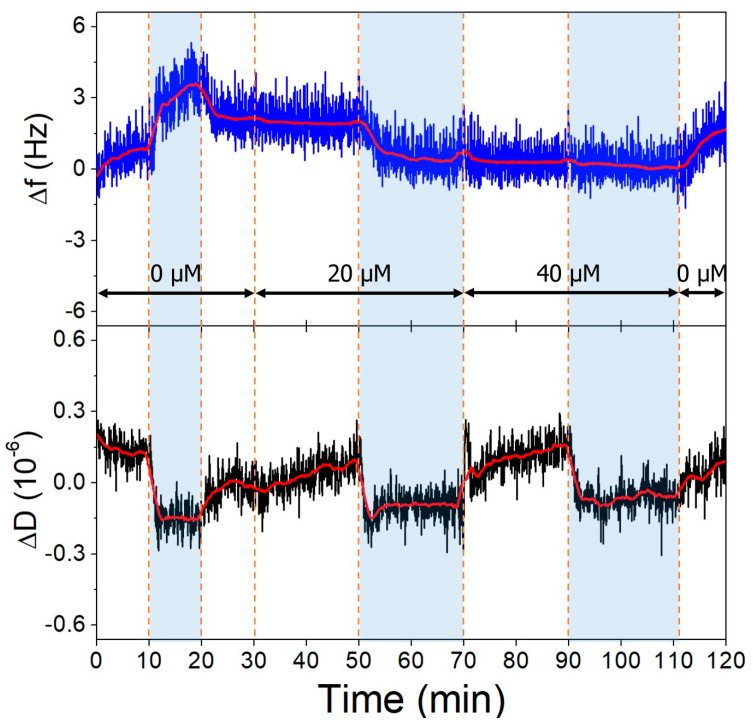
The third overtone frequency (**top**) and dissipation (**bottom**) variation values of low-aptamer-density gold surface in response to OTC. Dashed lines indicate solution changes, with white background corresponding to pH 7.1 and blue to pH 9.1. Different OTC concentrations are indicated in the figure.

**Figure 11:**
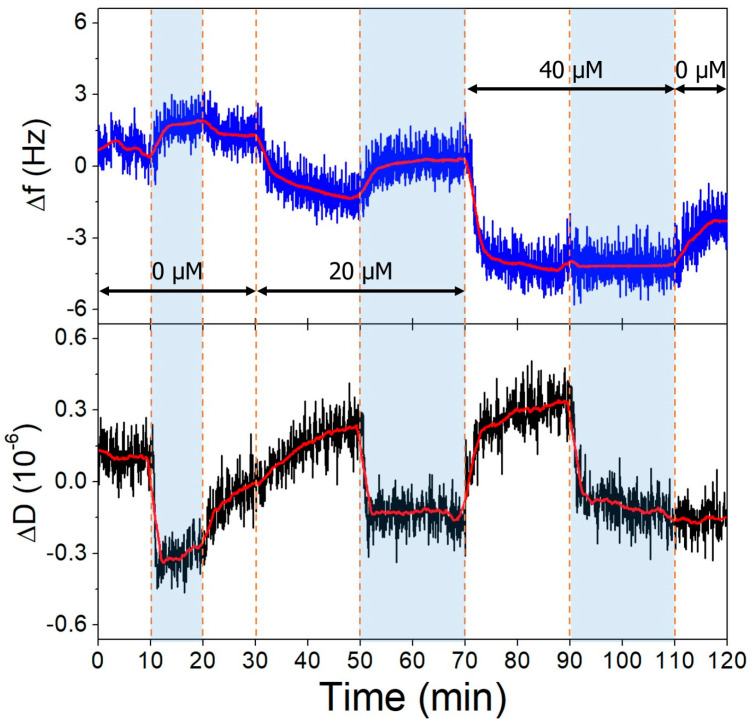
The third overtone frequency (**top**) and dissipation (**bottom**) variation values of high-aptamer-density gold surface in response to OTC. Dashed lines indicate solution changes, with white background corresponding to pH 7.1 and blue to pH 9.1. Different OTC concentrations are indicated in the figure.

The gold surfaces of the QCRs were modified with high and low aptamer concentrations and l-cysteine resulting in high- and low-coverage aptamer layers. These were subjected to increasing concentrations of OTC solutions, whereupon frequency changes indicative of aptamer-OTC interaction were observed. The addition of 20 µM OTC resulted in only a slight decrease at the low aptamer density surface at pH = 9.1 (Figure 10), which did not decrease further after increasing the concentration twofold, indicating that the aptamers are already saturated in the presence of 20 µM OTC. In the case of the high-aptamer-density surface (Figure 11), a larger frequency decrease was observed after the interaction with 20 µM OTC than with the low-density surface. This was followed by a further, proportionally larger decrease, when the 40 µM solution was introduced. These findings support the notion of preparing optimal aptamer surface densities for sensor applications [25,26,55].

Both on the low- and high-aptamer-coverage surfaces, the dissipation drops when the pH of the solutions is changed from 7.1 to 9.1. This change can be linked to the l-cysteine-based antifouling layer. The back and forth switching between pH conditions had little effect on the frequency baseline.

## 4. Conclusions

In this work, a comparative investigation of the pH- and target-dependent structural characteristics of an antibiotic-binding aptamer in dissolved and surface-bound form was presented. By using circular dichroism spectroscopy, the native and the antibiotic-bound conformation of the oxytetracycline aptamer was determined in solution: the aptamer assumes a random coil by itself, and the presence of oxytetracycline induces antiparallel G-quadruplex formation. Denaturation experiments further confirmed the loosely structured nature of the native aptamer and the high affinity and strong stabilizing effect of the target molecule. A significant effect of pH on the structure of the aptamer and the oxytetracycline–aptamer complex was observed. Protonation of DNA bases resulted in the formation of a more compact native aptamer structure in acidic buffers compared to the loosely ordered structure in neutral and alkaline solutions. Moreover, the interaction of the aptamer with the oxytetracycline target was affected in alkaline solutions: the deprotonation of bases prevented the guanine quadruplex’s formation. These phenomena were also demonstrated on the surface-immobilized aptamer via quartz crystal microbalance measurements. The frequency alteration of the resonator was attributed to aptamer –target binding, while decreases in dissipation could be related to the development of a compact aptamer structure. A pH-dependent oxytetracycline adsorption to the clean gold surface was also observed, which could be hindered by the deposition of an l-cysteine adlayer. While l-cysteine serves well as an antifouling agent, it imparts an additional pH sensitivity to the surface due to its zwitterionic nature. This study highlights the importance of comprehensive investigations on the structural changes in aptamers during the development process of effective aptasensors.

## Data Availability

The data presented in this study are available in this article.
